# Upregulation of *ADAM19* in dendritic cells is associated with immune features and lower C-peptide levels in type 1 diabetes mellitus

**DOI:** 10.1186/s13098-026-02170-8

**Published:** 2026-06-05

**Authors:** Lijun Chen, Changjian Yan, Chunling Huang, Wanrong Lin, Jinliang Xu, Yingxuan Lin, Hongli Lin, Zhengrong Jiang, Yong Zhuang, Huibin Huang

**Affiliations:** 1 Department of Endocrinology, The Second Affiliated Hospital of Fujian Medical University, No. 950 Donghai Street, Fengze District, Quanzhou City, 362000 Fujian Province China; 2 Department of Hematology, The Second Affiliated Hospital of Fujian Medical University, Quanzhou City, 362000 Fujian Province China; 3 Department of Electrocardiogram, The Second Affiliated Hospital of Fujian Medical University, Quanzhou City, 362000 Fujian Province China; 4 Department of Endocrinology, Shishi Municipal Hospital, Quanzhou City, 362700 Fujian Province China; 5 Department of Cardiology, Yongchun County Hospital, Quanzhou City, 362600 Fujian Province China; 6 The School of Clinical Medicine, Fujian Medical University, Fuzhou City, 350000 Fujian Province China

**Keywords:** *ADAM19*, T1DM, Dendritic Cells, Immune Microenvironment, Single-cell RNA Sequencing

## Abstract

**Background:**

Type 1 diabetes mellitus (T1DM) is a chronic metabolic disorder characterized by the autoimmune destruction of insulin-producing β-cells in the pancreas, leading to absolute insulin deficiency and persistent hyperglycemia. The immune system plays a pivotal role in the development of T1DM, with dendritic cells (DCs) acting as crucial modulators of immune responses. Despite this, the specific role of ADAM19 within the T1DM immune microenvironment remains poorly understood, particularly its expression in DCs and potential effects on pancreatic β-cell function. Further research is needed to explore these aspects in peripheral blood mononuclear cells (PBMCs) and pancreatic tissues.

**Objective:**

This study aims to investigate the association between ADAM19 expression in DCs and immune features in T1DM. Among the genes consistently upregulated across four independent PBMC transcriptomic datasets and previously implicated in immune regulation, ADAM19 exhibited the most pronounced differential expression in dendritic cell subsets. We therefore focused on ADAM19 to determine whether it influences DC function, thereby disrupting the immune microenvironment and exacerbating pancreatic β-cell damage. Additionally, the study evaluates the potential of ADAM19 as a candidate biomarker for T1DM.

**Methods:**

This study analyzed public bulk RNA-seq data from peripheral blood mononuclear cells (PBMCs) derived from three datasets (GSE156035, GSE55100, GSE9006), comprising a total of 129 samples (75 T1DM patients and 54 healthy controls). For discovery, an additional in-house PBMC RNA-seq cohort of 20 samples (13 T1DM, 7 controls) was included. Single-cell RNA-seq (scRNA-seq) data were obtained from PBMCs and pancreatic islets, comprising a total of 117 samples (55 from T1DM patients and 62 from healthy controls), with detailed distributions including 46 T1DM and 31 controls from PBMCs, and 9 T1DM and 31 controls from pancreas samples. By integrating transcriptomic and single-cell data, we evaluated the expression patterns of ADAM19 in DCs from T1DM patients. Analytical approaches included partial least squares discriminant analysis (PLS-DA), differential expression analysis (DEG), Gene Set Enrichment Analysis (GSEA), pseudotime trajectory analysis, and cell–cell communication inference to explore the potential involvement of ADAM19 in immune responses. For validation, an independent in-house cohort of PBMC samples (44 samples: 29 T1DM, 15 controls) was examined using RT-qPCR to assess the correlation between ADAM19 expression and C-peptide levels.

**Results:**

ADAM19 expression was consistently elevated in PBMCs of T1DM patients across all datasets, with the highest levels observed in DCs in single-cell RNA-seq data. Receiver operating characteristic (ROC) analysis demonstrated AUC values greater than 0.7 in all cohorts, reaching 0.93 in the in-house dataset. Elevated ADAM19 expression in DCs was associated with altered immune cell proportions and reduced fasting C-peptide levels in T1DM patients.

**Conclusion:**

This study identifies an association between elevated ADAM19 expression in DCs and T1DM immune features, suggesting potential links to immune dysregulation and β-cell dysfunction. ADAM19 shows promise as a candidate biomarker, warranting further validation for diagnostic and therapeutic applications.

**Supplementary Information:**

The online version contains supplementary material available at 10.1186/s13098-026-02170-8.

## Article highlights

1. *ADAM19* is significantly upregulated in DCs of peripheral blood mononuclear cells from patients with T1DM and accompanies the transition of DCs subtypes during disease progression.

2. Elevated *ADAM19* expression is associated with reduced pancreatic β-cell function and the immune microenvironment of T1DM.

3. *ADAM19* demonstrates potential as a biomarker in retrospective cohorts to distinguish T1DM patients from healthy controls.

**Supplementary Information:**

The online version contains supplementary material available at 10.1186/s13098-026-02170-8.

## Introduction

Type 1 diabetes mellitus (T1DM) is a long-term metabolic disorder marked by sustained high blood sugar and a complete lack of insulin, due to the immune system attacking insulin-producing pancreatic β-cells [1]. The global incidence of T1DM has risen steadily in recent decades. Although China has a lower incidence than Western countries [2], the large population means the total number of patients is still very high [3–5], This creates a significant public health burden. The development of T1DM results from the complex interplay of various elements, including genetic predisposition (e.g., MHC class II variants), environmental exposures, and dysregulated immune responses. It is generally accepted that autoreactive T lymphocytes, activated by antigen-presenting cells such as dendritic cells (DCs), infiltrate the pancreatic islets and initiate an inflammatory cascade, ultimately leading to the gradual destruction of insulin-producing β-cells. Nevertheless, the fundamental molecular events that trigger the autoimmune response are not yet fully elucidated, and reliable biomarkers closely linked to disease progression are still lacking, which significantly hampers the advancement of early intervention approaches [6, 7].

In recent years, growing interest has focused on how the immune microenvironment influences the development of T1DM. In addition to T lymphocytes, other immune cells—including natural killer (NK) cells [8], B lymphocytes [9] and DCs [9, 10]—contribute to disease progression by releasing cytokines such as IL-12 and IFN-α, as well as through antigen presentation. DCs serve as a crucial bridge linking innate and adaptive immunity in T1DM. They capture, process, and present autoantigens originating from pancreatic β-cells, while simultaneously regulating T cell responses by producing cytokines including IL-12, IFN-α, and IL-10, thereby maintaining a balance between immune activation and suppression [11]. Importantly, early therapeutic strategies aimed at DCs carry significant clinical value, since once autoreactive T cells become activated and begin attacking β-cells, establishing immune tolerance afterward is exceedingly challenging [12]. Thus, thoroughly exploring the regulatory pathways controlling DC function is essential for understanding T1DM pathogenesis and identifying new treatment strategies.

This study hypothesizes that elevated *ADAM19* expression may be associated with alterations in the pancreatic islet immune microenvironment via DCs. *ADAM19* (A Disintegrin And Metalloproteinase 19, also known as meltrin β), a member of the ADAM protein family, has emerged as a potential regulator of DC function and may represent a promising therapeutic target. This multifunctional protease is composed of several distinct domains, including a prodomain, a catalytic metalloproteinase region, and a disintegrin-like domain [13]. Its metalloproteinase domain contributes to extracellular matrix remodeling and immune signaling by cleaving membrane-bound substrates, such as TNF-α, thereby facilitating ectodomain shedding [14]. *ADAM19* is broadly expressed in peripheral blood mononuclear cells and metabolically active tissues, such as the liver [14]. It has been implicated in cardiovascular development [15], skeletal muscle remodeling [16], and the pathophysiology of metabolic syndrome [17], and also contributes to the progression of autoimmune disorders [13]. Nevertheless, the expression profile of *ADAM19* in T1DM and its potential role in disrupting the pancreatic islet immune microenvironment by regulating DC function remain poorly understood. While *ADAM19* has been implicated in autoimmune disorders, its expression in T1DM DCs and potential associations with β-cell function remain underexplored.

Building on this background, the present study hypothesizes that *ADAM19* may be involved in the inflammatory microenvironment within pancreatic islets and β-cell injury, potentially through its modulation of DC-driven antigen presentation or T cell infiltration. To test this hypothesis, the study combines transcriptome sequencing data from 149 clinical samples (88 patients with T1DM and 61 healthy controls) and single-cell sequencing data from 117 samples (55 T1DM patients and 62 healthy controls) to comprehensively profile *ADAM19* expression in affected individuals. Furthermore, the research assesses its potential as a candidate biomarker examines its correlation with immune cell dysfunction, and explores its involvement in regulating the transition among DC subpopulations. The results of this study are anticipated to offer novel understanding of the immunopathology underlying T1DM and to establish a theoretical basis for early diagnosis and the development of targeted therapies centered on *ADAM19*.

## Materials and methods

### RNA-seq sequencing

#### Sample collection

The in-house dataset analyzed in this research was collected from the Endocrinology Department at the Second Affiliated Hospital of Fujian Medical University, encompassing patients admitted from January 2024 to March 2025. The dataset comprises 13 individuals diagnosed with T1DM and 7 healthy controls matched by age and sex. All specimens were collected as whole blood samples. Diabetes diagnosis followed the World Health Organization (WHO) 1999 guidelines, requiring typical clinical symptoms such as excessive thirst, hunger, urination, or weight loss, along with at least one of the following criteria: random plasma glucose ≥ 11.1 mmol/L; fasting plasma glucose ≥ 7.0 mmol/L; or a 2-hour plasma glucose ≥ 11.1 mmol/L measured during a 75-gram oral glucose tolerance test (OGTT). If diabetic symptoms do not appear, repeated blood glucose assessments are necessary to confirm the diagnosis. The inclusion criteria for T1DM included sudden disease onset, a predisposition or prior episode of diabetic ketoacidosis, continuous insulin dependency, and either the presence of one or more islet-specific autoantibodies or C-peptide concentrations below the reference range. Participants in the healthy control group were required to exhibit fasting glucose levels under 6.1 mmol/L and post-meal glucose levels below 7.8 mmol/L. Individuals were excluded if they had coexisting autoimmune disorders, atypical forms of diabetes, were pregnant or breastfeeding, or had acute or chronic infections, recent trauma, surgical history, physiological stress, hepatic or renal impairment, malignant tumors, psychiatric illness, or known infectious diseases. Informed consent was obtained in both oral and written form from all participants before inclusion. The study protocol was reviewed and approved by the Ethics Committee of the Second Affiliated Hospital of Fujian Medical University (2025 Ethics Approval No. 100, The Second Affiliated Hospital of Fujian Medical University).

#### Isolation of peripheral blood mononuclear cells (PBMCs)

Two milliliters of peripheral blood collected in EDTA-treated tubes were combined with an equal volume of phosphate-buffered saline (PBS) for subsequent processing. A volume of 2 mL lymphocyte separation medium was added to a sterile 15 mL centrifuge tube. The previously diluted blood was gently layered above the medium to avoid disrupting the interface, then centrifuged at 2300 rpm for 20 min at 23 °C. Following centrifugation, the sample stratified into four distinct layers: plasma, the lymphocyte layer, the separation medium, and a cell pellet at the tube’s base. The lymphocyte layer was gently collected and washed with PBS at three times its volume, then centrifuged at 1800 rpm for 15 min at 23 °C. After discarding the supernatant, red blood cell lysis buffer was added and mixed thoroughly, followed by centrifugation at 1500 rpm for 10 min at 23 °C. Following a second PBS wash, the cell pellet was resuspended in TRIzol reagent and preserved at -80 °C.

#### RNA isolation and quality assessment

RNA was isolated utilizing the PAXgene Blood miRNA Kit. Concentration measurements were performed with a Nanodrop 2000 spectrophotometer, while RNA integrity was evaluated through Agilent 2100 Bioanalyzer and LabChip GX platforms. RNA samples were considered qualified if they met the following standards: total yield of at least 2 µg, concentration above 40 ng/µL, OD260/280 ratio ranging from 1.7 to 2.5, OD260/230 ratio between 0.5 and 2.5, and an RNA integrity number (RIN) equal to or greater than 7.

#### Library construction method

Initially, mRNA was isolated with mRNA Capture Beads and incubated at 65 °C to promote attachment to the magnetic beads. Next, the mRNA was isolated using a magnetic stand, eluted with Tris buffer, and subsequently fragmented. Then, complementary DNA (cDNA) was synthesized through reverse transcription, after which the cDNA was purified. Following end repair, adapters containing index sequences were ligated to enable library construction. After library amplification, impurities were eliminated using magnetic beads, and the final product was recovered by elution with nuclease-free water. Ultimately, library quality was evaluated using the Qsep-400 system to confirm its suitability for sequencing.

#### Sequencing method

High-throughput sequencing was conducted on the Illumina NovaSeq X Plus platform employing paired-end 150 bp (PE150) mode.

### Reverse transcription quantitative PCR (RT-qPCR)

#### Sample collection

PCR samples used in this study were collected from patients who received inpatient or outpatient care at the Endocrinology Department at the Second Affiliated Hospital of Fujian Medical University between January 2024 and March 2025. The dataset comprises 29 individuals diagnosed with T1DM and 15 healthy controls matched for age and sex. All samples were collected as whole blood specimens. The inclusion and exclusion standards applied to this cohort were identical to those outlined previously. All participants were given both oral and written explanations of the study and signed informed consent forms before enrollment. The research protocol received approval from the Ethics Committee of the Second Affiliated Hospital of Fujian Medical University (2025 Ethics Approval No. 100, The Second Affiliated Hospital of Fujian Medical University).

#### RT-qPCR experimental method

The procedure and reagents used for PBMC isolation followed the previously established protocol. cDNA synthesis was carried out using SynScript^®^ III RT SuperMix for qPCR. Primers specific to *ADAM19* and β-actin genes were designed utilizing the NCBI Primer-BLAST. The PCR reaction mixture was composed of TB Green Premix EX Taq II, Rox Reference Dye or Dye II, forward and reverse primers, DEPC-treated water, and reverse transcription (RT) reaction solution, adjusted to a final volume of 10.0 µL. qPCR was carried out using the MA-6000 system under these cycling parameters: an initial denaturation at 95 °C for 5 min, followed by 40 cycles of 95 °C for 15 s, 60 °C for 20 s, and 72 °C for 20 s. Data were analyzed using the 2^*−ΔΔC*t^ approach, normalizing target gene expression to β-actin as the internal control. All samples were tested in triplicate to guarantee technical consistency.

### Collection and processing of external bulk transcriptome data

Transcriptomic data of PBMCs were obtained from 75 individuals diagnosed with T1DM and 54 healthy controls. The data were retrieved from three datasets in the Gene Expression Omnibus (GEO) database (http://www.ncbi.nlm.nih.gov/geo/): GSE156035, GSE55100, and GSE9006. The GSE156035 dataset comprises 40 samples, evenly split between 20 T1DM patients and 20 healthy controls. The GSE55100 dataset contains 22 samples, including 12 T1DM individuals and 10 healthy participants. Meanwhile, the GSE9006 dataset consists of 67 samples, with 43 derived from T1DM patients and 24 from healthy controls. All included studies adhered to the respective ethical review guidelines.

Each bulk transcriptomic dataset (GSE156035, GSE55100, GSE9006, and in-house RNA-seq) was analyzed independently to serve as a cross-cohort replication strategy. No cross-dataset merging or combined batch correction was performed for the bulk data. Instead, consistent preprocessing and statistical methods were applied across all four datasets to ensure comparability of results.

### Partial least squares discriminant analysis (PLS-DA) and differential gene expression analysis

Further analyses were performed independently on each dataset. PLS-DA was carried out utilizing the ropls package (version 1.38.0) within the R programming language. The scores of the Principal Component 1 (PC1) and Principal Component 2 (PC2) were utilized to create a two-dimensional plot, with samples color-coded by group and confidence ellipses depicting intra-group variability. The plots were created utilizing the ggplot2 package. Differentially expressed genes (DEGs) between T1DM patients and healthy controls were detected using the wilcoxauc function from the presto package (version 1.0.0) in R. Genes exhibiting p-values less than 0.05 and average log_2_ fold change exceeding 0.1 were classified as significantly upregulated. Significantly differentially expressed genes were illustrated through boxplots created using the ggplot2 package. Receiver operating characteristic (ROC) curves were constructed with the pROC package (version 1.18.5) in R to assess the discriminative performance of *ADAM19* between individuals with T1DM and healthy controls.

### Collection and processing of single-cell RNA sequencing (scRNA-seq) data

This study utilized single-cell transcriptomic data obtained from two publicly accessible sources. The first dataset comprised scRNA-seq data of PBMCs from 46 individuals diagnosed with T1DM and 31 healthy controls, accessed through the Synapse platform (ID: syn53361982) [18]. The second dataset consisted of pancreatic scRNA-seq results from 9 T1DM patients and 31 healthy controls, retrieved from the Human Pancreas Analysis Program (HPAP; RRID: SCR_016202, https://hpap.pmacs.upenn.edu). Routine downstream analysis was carried out with Scanpy v1.11.1. Quality control filtering retained cells exhibiting mitochondrial gene expression below 15%, total counts exceeding 1000, and gene counts greater than 500. Doublets were predicted employing the Scrublet al.gorithm under default settings. In total, 217,084 high-quality cells were retained for analysis. Batch effect correction was conducted using the Harmony algorithm, applying a clustering resolution parameter of 2. Visualization in two-dimensional space was performed using Uniform Manifold Approximation and Projection (UMAP). Cell types were annotated using established marker genes characteristic of each population.

## Analysis of cell-cell communication

Cell-cell communication patterns across various conditions were assessed using the multinichenetr R package (version 2.1.0). Adhering to the MultiNicheNet recommended workflow, the following procedures were conducted:

### Cell type selection

number of cells corresponding to each defined cell type was quantified separately for each condition. Only cell types meeting a minimum threshold (e.g., at least five cells per group) were included for subsequent analysis. Cell distribution plots were generated to visually represent the filtered populations.

### Gene expression filtering

An expression cutoff was applied based on the fraction of cells expressing each gene (fraction_cutoff = 0.1), retaining only those genes significantly expressed in at least one condition for downstream analyses.

### Pseudo-bulk expression calculation

Expression data from all cells within each cell type were aggregated and normalized to construct a sample-level expression matrix.

### Differential expression analysis

Differential expression analysis comparing conditions was conducted using the FindMarkers function to detect ligands, receptors, and candidate target genes, with corresponding log fold changes (logFC) and significance values documented.

### Inference of ligand activity and downstream target prediction

Based on a curated ligand–receptor–target gene interaction network, ligands exhibiting regulatory activity within each receiver cell type were predicted, and their principal downstream target genes were inferred accordingly.

### Prioritization of ligand–receptor interaction

A multidimensional weighted ranking strategy was employed to prioritize ligand–receptor interactions, integrating ligand expression abundance, differential expression metrics, and predicted activity scores, thereby characterizing key intercellular signaling pathways under both pathological and physiological states.

### Gene set enrichment analysis (GSEA)

Functional enrichment analysis targeting Gene Ontology Biological Process (GOBP) gene sets sourced from the Molecular Signatures Database (MSigDB) was carried out using the clusterProfiler package (version 4.12.6). The enrichment outcomes were subsequently visualized.

### Monocle 2 trajectory analysis

To explore the association between *ADAM19* and the differentiation of DC subtypes, cellular developmental potential was quantified using the CytoTRACE2 R package (version 1.1.0). The subtype exhibiting the greatest developmental potential was designated as the root for subsequent trajectory inference. Data preprocessing, including filtering and normalization, along with trajectory inference—comprising dimensionality reduction and minimum spanning tree construction—was performed using the monocle2 R package (version 2.32.0) with default settings. Trajectory visualization was achieved using UMAP or t-SNE, followed by gene expression clustering analysis.

### Statistical analysis

All statistical analyses were performed using R software (version 4.4.1). Normality of continuous variables was assessed using the Shapiro–Wilk test. For variables following a normal distribution, comparisons between two groups were conducted using independent samples t-tests, while one-way analysis of variance (ANOVA) was used for comparisons involving multiple groups. For variables violating normality assumptions, group differences were evaluated using the Mann–Whitney U test for two-group comparisons and the Kruskal–Wallis test for multiple-group comparisons. Categorical variables were summarized as frequencies and percentages (%), and differences in proportions between groups were assessed using the chi-square (χ²) test. A two-tailed significance level of α = 0.05 was applied, with *P* < 0.05 considered statistically significant.

Differential expression analysis for bulk RNA-seq data was performed using the Wilcoxon rank-sum test. Genes with an adjusted *P* < 0.05 and |log₂ fold change| ≥ 0.25 were considered differentially expressed. The same test and thresholds were applied to single-cell RNA-seq differential expression analysis. P-values were adjusted for multiple comparisons using the Benjamini–Hochberg method. Gene set enrichment analysis (GSEA) was conducted using a permutation-based approach, with significantly enriched gene sets defined as those with an adjusted *P* < 0.05. Gene Ontology (GO) enrichment analysis was performed using a hypergeometric test, and significant terms were identified at an adjusted *P* < 0.05. For pseudotime analysis, genes associated with pseudotime were identified using a likelihood ratio test, with significance determined at a more stringent threshold of adjusted *P* < 0.01 to account for the large number of genes tested along the trajectory.

## Results

### ADAM19 as a potential T1DM biomarker

The clinical characteristics of participants across all cohorts are detailed in Supplementary Table S1. In brief, the in-house RNA-seq cohort (13 T1DM patients and 7 controls) and RT-qPCR cohort (29 T1DM patients and 15 controls) were well-matched for age and sex between T1DM and healthy control groups (age: 48.8 ± 15.9 vs. 47.1 ± 9.9 years and 43.0 ± 18.1 vs. 45.7 ± 9.0 years, respectively). As expected, HbA1c levels were markedly elevated and fasting C-peptide levels were significantly reduced in T1DM patients, consistent with β-cell dysfunction. The GEO datasets provided limited clinical details but aligned with typical pediatric/adult T1DM profiles reported in the literature.

**Fig. 1:**
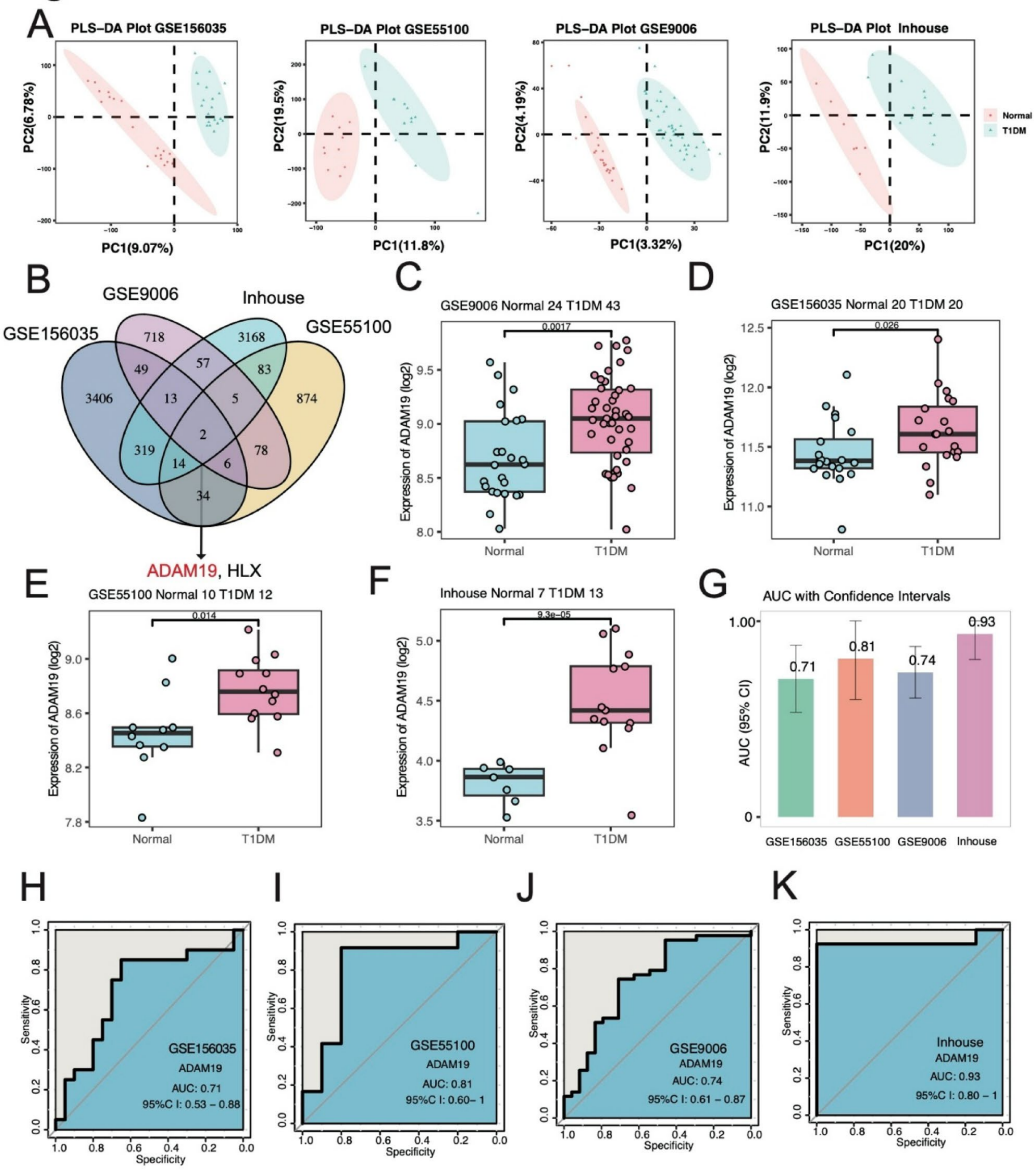
Expression and Predictive Capability Analysis of *ADAM19* in PBMC from Different Datasets. **A**. Partial Least Squares Discriminant Analysis (PLS-DA) plot showing the distribution differences between the normal (*n* = 61) and T1DM (*n* = 88) groups in the GSE156035, GSE55100, GSE9006, and in-house PBMC datasets. **B**. Venn diagram displaying the intersection of upregulated genes, including *ADAM19*, across the four PBMC datasets. **C**-**F**. Box plots in each PBMC dataset illustrating the expression level differences of *ADAM19* between healthy controls and T1DM patients (GSE156035: 20 T1DM vs. 20 controls; GSE55100: 12 T1DM vs. 10 controls; GSE9006: 43 T1DM vs. 24 controls; in-house: 13 T1DM vs. 7 controls). **G**. *ADAM19* predictive ability evaluation across PBMC datasets, showing the AUC values and their 95% confidence intervals. **H**-**K**. ROC curve analysis assessing the diagnostic performance of *ADAM19* in PBMC in each dataset (GSE156035: *n* = 40; GSE55100: *n* = 22; GSE9006: *n* = 67; in-house: *n* = 20)

Transcriptomic data (Fig. 1A) were collected from PBMCs, comprising 149 individual samples including 88 T1DM patients and 61 healthy controls, sourced from in-house datasets and public repositories (GSE156035, GSE55100, GSE9006). In the PLS-DA plot of the GSE156035 dataset, PC 1 (9.07%) and PC 2 (6.78%) demonstrated distinct separation between groups, reflecting significant differences between the healthy control and T1DM cohorts. Comparable group separation patterns were observed in the PLS-DA analyses of the GSE55100 (PC 1: 11.8%, PC 2: 19.5%) and GSE9006 (PC 1: 3.32%, PC 2: 4.19%) datasets, reflecting consistent variation across sample groups. The in-house dataset ultimately exhibited a clear separation between control and T1DM samples along the PC 1 (20%) and PC 2 (11.9%). The in-house dataset revealed similar separation patterns. The results indicated notable and consistent transcriptomic variation between healthy control and T1DM groups.

As shown in Fig. 1B, two genes—*ADAM19* and *HLX*—were consistently upregulated across all four datasets. Among these, *ADAM19* exhibited the most pronounced differential expression in DC subsets and showed the strongest association with immune features of T1DM.Additionally, previous studies have implicated *ADAM19* in immune regulation and autoimmune diseases, making it a compelling candidate for further investigation. This gene exhibited marked elevation in the GSE156035, GSE55100, GSE9006, and in-house cohorts (Fig. 1B; Tables S2–S5). Given its consistent upregulation across the analyzed datasets and its largely unexplored involvement in T1DM, this study focused on *ADAM19* to examine its potential contributions to disease development and progression.

*ADAM19* expression was further compared between 54 healthy individuals and 75 T1DM patients, revealing a significant elevation in the T1DM cohort compared to controls. In the GSE9006 dataset, *ADAM19* expression was significantly higher in the T1DM group (*n* = 43) than in healthy controls (*n* = 24) (t-test, *p* = 0.0017, Fig. 1C). Likewise, the GSE156035 dataset showed marked upregulation of *ADAM19* in T1DM patients (*n* = 20) compared to controls (*n* = 20) (t-test, *p* = 0.026, Fig. 1D). In the GSE55100 dataset, *ADAM19* expression differed significantly between the T1DM group (*n* = 12) and healthy controls (*n* = 10) (t-test, *p* = 0.014, Fig. 1E). Our in-house transcriptomic sequencing dataset also yielded consistent results: *ADAM19* levels were significantly higher in T1DM patients (*n* = 13) compared to healthy subjects (*n* = 7) (t-test, *p* = 9.31 × 10⁻⁵, Fig. 1F). The persistent and significant elevation of *ADAM19* observed across several datasets indicates a likely key involvement in T1DM pathogenesis, reinforcing its promise as a candidate biomarker.

ROC curve analysis was conducted to assess the diagnostic performance of *ADAM19* in differentiating T1DM patients from healthy controls. Results indicated that *ADAM19* exhibited suggests potential utility in retrospective cohorts in all datasets, with area under the curve (AUC) values above 0.7 throughout (Fig. 1G). Specifically, the AUC values were 0.71 (95% CI: 0.53–0.88) for GSE156035, 0.81 (95% CI: 0.61–1.00) for GSE55100, 0.74 (95% CI: 0.61–0.87) for GSE9006, with the highest AUC of 0.93 (95% CI: 0.80–1.00) observed in the in-house dataset (Fig. 1H-K**)**. Despite variability in AUC values among datasets, the collective findings demonstrate that *ADAM19* possesses strong diagnostic accuracy, effectively differentiating healthy individuals from T1DM patients.

### DC–specific upregulation of ADAM19 in the T1DM immune microenvironment

**Fig. 2:**
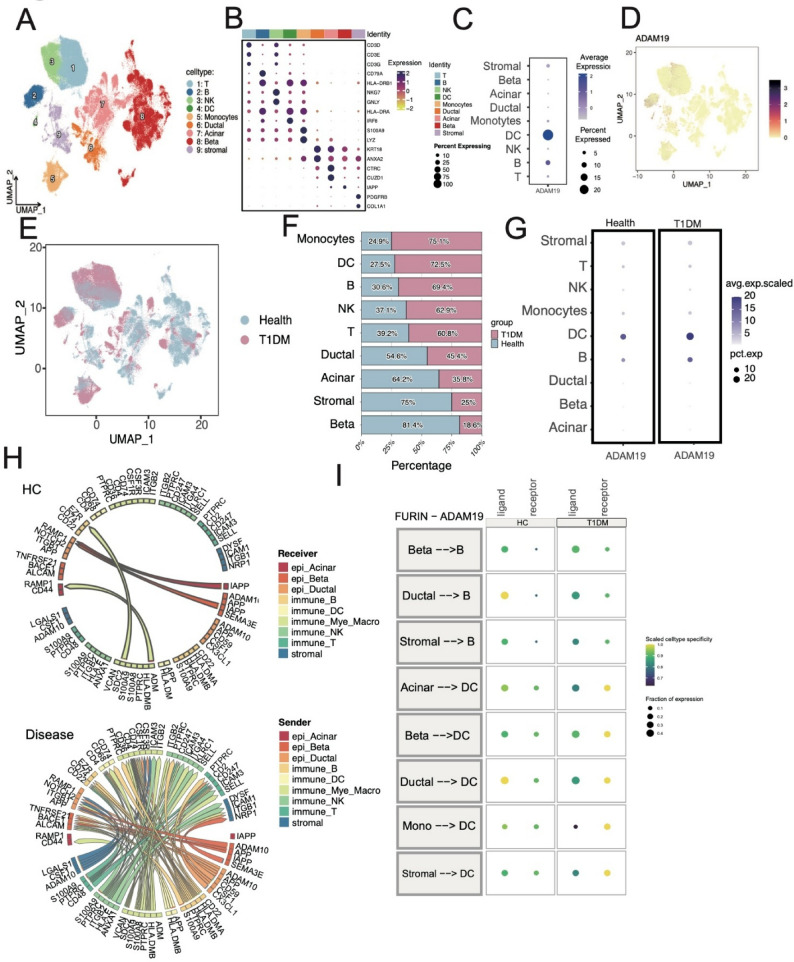
Cell Type Distribution and Expression Analysis of *ADAM19* in PBMC and Pancreatic Islets of T1DM Patients. **A**. UMAP dimensionality reduction plot showing the distribution of cell populations in healthy controls (*n* = 62) and T1DM patients (*n* = 55) from PBMC and pancreatic islets. **B**. Dot plot canonical marker genes for each cell type. **C**. Violin plot showing *ADAM19* expression levels across different cell types. **D**. UMAP plot showing the expression distribution of *ADAM19* gene (highlighting enrichment in DCs). **E**. UMAP dimensionality reduction plot comparing cell population distributions between healthy controls and T1DM groups (PBMC and pancreatic islets). **F**. Stacked bar chart showing the proportion of each cell type in healthy controls versus T1DM patients (PBMC and pancreatic islets). **G**. Comparison of *ADAM19* expression levels in different cell types between healthy controls and T1DM patients (PBMC and pancreatic islets). **H**. Heatmap plot of overall cell-cell communication strength in the immune microenvironment between healthy and T1DM groups (PBMC and pancreatic islets). **I**. Ligand-receptor interaction analysis focusing on the *FURIN-ADAM19* pair, showing interaction strength between different cell types in healthy controls versus T1DM patients (PBMC and pancreatic islets)

ScRNA-seq data from 117 samples (62 healthy controls and 55 T1DM patients) were collected and analyzed, including 46 T1DM and 31 healthy controls from PBMCs, and 9 T1DM and 31 healthy controls from pancreatic samples. Following rigorous quality filtering, 217,084 cells meeting quality criteria were retained for analysis. Cells were clustered into nine distinct types, with canonical marker genes displayed as follows: T cells identified by CD3D, CD3E, and CD3G; B cells by CD79A [19]; NK cells marked by NKG7 and GNLY [20]; DCs characterized by HLA-DRB1 and IRF8 [21]; monocytes expressing S100A9 and LYZ [22]; ductal cells marked with KRT18 and ANXA2 [23]; acinar cells indicated by CTRC, CUZD1, and IAPP [24];β cells identified by IAPP [25]; and stromal cells marked by PDGFRB and COL1A1 [26] (Fig. 2A-B, **Table S6**). Subsequent analysis demonstrated that *ADAM19* expression is markedly elevated in DCs. UMAP visualization confirmed that *ADAM19* expression is predominantly enriched within the DC cluster. These results indicate that *ADAM19* is selectively overexpressed in DCs, implying a possible functional involvement in this cell type (Fig. 2C-D).

UMAP analysis revealed distinct group-specific distribution patterns between healthy controls and T1DM patients despite partial overlap, particularly for T cells, B cells, NK cells, DCs, and monocytes(Fig. 2E). A comparative assessment of cell type proportions was performed to investigate distributional differences between healthy individuals and T1DM patients. While monocytes showed high proportions in T1DM (75.1%), this may reflect dataset-specific composition (e.g., insulitis in pancreas data) and has not been universally reported in PBMC-only studies.The analysis demonstrated a marked increase in monocyte proportion in the T1DM group (75.1%) relative to the healthy controls (24.9%). Likewise, DCs and B cells were more prevalent among individuals with T1DM, comprising 72.5% and 69.4% of their respective populations, in contrast to lower proportions in the control group. Furthermore, NK cells and T cells exhibited significantly increased proportions in the T1DM group compared to healthy controls. Such alterations are strongly linked to the immune-inflammatory processes underlying T1DM, indicating an enhanced involvement of immune cells in disease progression. Conversely, β cells constituted a significantly larger proportion in healthy individuals (81.4%) than in T1DM patients (18.6%), reflecting substantial β cell depletion associated with the disease. This observation reinforces the pivotal role of beta cell loss in T1DM development (Fig. 2F). Expression levels of the *ADAM19* gene were compared between individuals in the healthy control group and those diagnosed with T1DM. The results revealed variable *ADAM19* expression among different cell types, with significantly elevated levels observed in DCs from T1DM patients relative to healthy individuals (Fig. 2G).

To address potential compositional confounding, we further evaluated the cell-type specificity of *ADAM19* expression across all 217,084 cells (Figure S1). The results showed that *ADAM19* was highly enriched in DCs, with 24.5% of DCs expressing the gene (Figure S1A). Compared with monocytes/macrophages, the proportion of cells expressing *ADAM19* in DCs was 9.4-fold higher (2.6% in monocytes/macrophages). The apparently high monocyte proportion (75.1%) observed in the merged dataset was an artifact resulting from the combined analysis of PBMC and pancreatic samples. Stratified analysis revealed that monocyte proportions in PBMC samples fell within the normal ranges reported in the literature (healthy controls: 7.59%; T1DM: 14.31%), whereas monocyte proportions in pancreatic samples remained consistently low (healthy controls: 0.75%; T1DM: 1.32%) (Figure S1B-C). More critically, within-cell-type differential expression analysis demonstrated that the upregulation of *ADAM19* in T1DM patients was most pronounced in DCs (Figure S1D, *p* = 0.0025), whereas no significant upregulation was observed in monocytes/macrophages (Figure S1D, *p* = 0.057; Figure S1E, *p* = 0.4). Although nominal statistical significance was also reached in some other cell lineages (e.g., B cells and T cells), the magnitude of change and baseline expression levels were substantially lower than those in DCs (Figure S1D–E). These findings indicate that the observed *ADAM19* signal is predominantly derived from DCs and is not driven by shifts in monocyte abundance. Collectively, our results suggest that *ADAM19* may play a critical role in the immune microenvironment of T1DM by modulating DC function.

Intercellular communication is essential for immune cell activation and chemotaxis. Such interactions can cause aberrant immune activation, which gradually intensifies and contributes to disease initiation and progression. We investigated the cellular interactions occurring within the immune microenvironment of healthy subjects and individuals with T1DM. The findings indicated (Fig. 2H, Table S7) a marked increase in intercellular communication within the T1DM group, especially among immune cells, highlighting elevated signaling dynamics involved in the disease’s immune response. Additional investigation demonstrated that(Fig. 2I) the *FURIN*-*ADAM19* ligand-receptor pair, incorporating *ADAM19*, is broadly involved in mediating intercellular communication. Among these, DCs, serving as receptor cells, displayed the greatest variety of interaction types. In contrast to the healthy group, DCs in the T1DM group had much stronger *ADAM19*-linked receptor interactions with acinar cells, pancreatic β cells, ductal cells, monocytes, and stromal cells. This suggests that the elevated *ADAM19* expression in DCs is crucial for changes in the immune microenvironment associated with T1DM .

### ADAM19 expression across DC subtype in T1DM

**Fig. 3:**
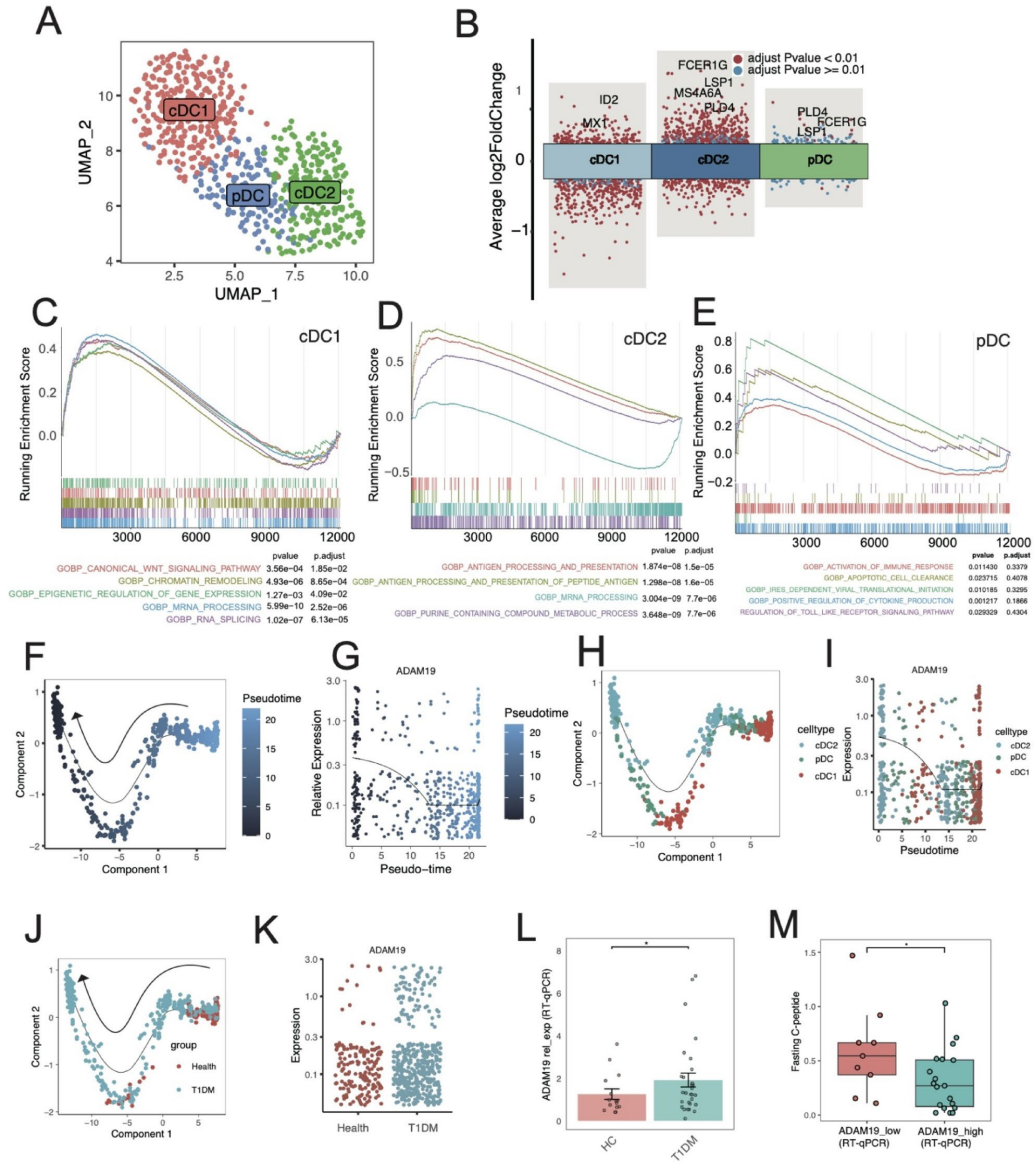
Expression of *ADAM19* and Its Impact on DC Subsets in T1DM (PBMC and Pancreatic Islets). **A**. UMAP dimensionality reduction plot showing the distribution of three DC subsets (cDC1, cDC2, and pDC) in PBMC and pancreatic islets. **B**. Differential gene expression analysis of the three DC subsets (cDC1, cDC2, pDC), highlighting significantly changed genes. **C**-**E**. Gene Set Enrichment Analysis (GSEA) plots for each DC subset (C: cDC1; D: cDC2; E: pDC) showing significantly enriched biological pathways. F. Pseudotime trajectory of DCs colored by DC subsets. **G**. Pseudotime trajectory showing the expression dynamics of *ADAM19* along the trajectory. **H**. Pseudotime trajectory colored by DC subsets (cDC1, cDC2, pDC). **I**. Expression of *ADAM19* along pseudotime in the three DC subsets. **J**. Pseudotime trajectory comparing healthy controls and T1DM patients. **K**. *ADAM19* expression dynamics along pseudotime in healthy controls versus T1DM patients (PBMC and pancreatic islets). **L**. RT-qPCR validation of *ADAM19* expression in PBMC from healthy controls (HC, *n* = 15) and T1DM patients (*n* = 29). **M**. Fasting C-peptide levels in T1DM patients stratified by low versus high *ADAM19* expression in PBMC (t-test, *p* < 0.05)

To further clarify the role of *ADAM19* in DC subtypes, we classified the DC population into three distinct subsets: type 1 conventional DCs (cDC1), type 2 conventional DCs (cDC2), and plasmacytoid DCs (pDCs) (Fig. 3A). In cDC1 cells, genes related to antigen presentation and immune activation, such as *ID2* and *MX1*, were significantly upregulated. In contrast, cDC2 cells exhibited higher expression of genes involved in cell migration and immune regulation, including *FCER1G*, *LSP1*, *MS4A6A*, and *PLD4*, with *FCER1G* displaying the most pronounced increase within this subtype. The pDC subset primarily showed increased expression of *FCER1G* and *LSP1*, genes implicated in antiviral defense and the regulation of immune tolerance (Fig. 3B, Table S8). GSEA revealed that the upregulated differentially expressed genes in cDC1 were mainly concentrated in pathways associated with immune responses, including “antigen presentation” and “cytokine signaling” (Fig. 3C, Table S9). By comparison, cDC2 cells demonstrated enrichment in gene sets involved in immune modulation, lymphocyte activation, and antiviral defense mechanisms (Fig. 3D, Table S10). The pDC subtype was mainly enriched in pathways linked to viral defense and antigen processing (Fig. 3E, Table S11). The pseudotime analysis suggests *ADAM19* expression increases along modeled trajectories, consistent with potential state transitions, though this is in silico modeling and not direct observation.

Subsequently, we examined the role of *ADAM19* in regulating transitions between DC subtypes by applying pseudotime trajectory analysis. We observed that *ADAM19* levels gradually increased along the pseudotime trajectory (Fig. 3F-G). Mapping the trajectory onto each subtype revealed that *ADAM19* expression rose as cells transitioned from cDC1 to pDC and then to cDC2. Moreover, *ADAM19* levels were higher in disease compared to healthy states. These findings suggest that *ADAM19* is important both for disease progression and for observed alongside changes among DC subtypes (Fig. 3H-K).

### Elevated ADAM19 in T1DM correlates with impaired pancreatic β-cell function

To assess the impact of *ADAM19* on pancreatic islet function in T1DM, we gathered 44 samples, consisting of 15 healthy controls and 29 T1DM patients. RT-qPCR analysis revealed that *ADAM19* expression was significantly higher in the disease group compared to the control group (t-test, *p* < 0.05, Fig. 3L). Further analysis on the effect of varying *ADAM19* expression levels on pancreatic β-cell function in T1DM patients showed that those with high *ADAM19* expression had notably lower fasting C-peptide levels (t-test, *p* < 0.05, Fig. 3M).

## Discussion

T1DM is a chronic autoimmune disorder marked by the destruction of pancreatic β-cells, resulting in inadequate insulin production. Although genetic and environmental factors both contribute to the onset of T1DM, the immune system’s role in the progression of the disease remains a pivotal area of investigation. DCs are key antigen-presenting cells (APCs) that play a central role in bridging the innate and adaptive immune systems. They are involved in a range of crucial functions, such as activating naive T cells and promoting their differentiation into effector and memory cells [27]. Moreover, DCs are essential players in the inflammatory response.

Typically, immature conventional cDCs are found in the islets. In the absence of danger or inflammatory signals, these DCs carry islet-derived antigens to the pancreatic lymph nodes (LNs), where they promote T cell tolerance. In contrast to the islets of non-diabetic prone mice, the islets of NOD mice begin to show infiltration by pDCs, B1a B cells, and neutrophils starting at two weeks of age. At two weeks of age, normal β-cell death results in the accumulation of numerous apoptotic β-cells. However, in NOD mice, these cells are not efficiently cleared. This, in turn, triggers B1a B cells to produce anti-DNA IgG, while neutrophils release CRAMP. These complexes bind to pDCs via the TLR9 receptor, triggering the production of significant amounts of IFN-α. IFN-α facilitates the maturation of cDCs infiltrating the islets, which subsequently activate islet-reactive CD4^+^ and CD8^+^ T cells in the draining lymph nodes. This triggers an autoimmune response and amplifies it through the ensuing local inflammation [10].

*ADAM19* is a recently identified metalloprotease that plays a crucial regulatory role in several immune-related diseases. In patients with rheumatoid arthritis (RA), DCs display a pronounced pro-inflammatory phenotype and demonstrate heightened responsiveness to TNF stimulation. Single-cell multi-omics analysis showed that in RA patients, the transcriptional programs activated by TNF stimulation are strongly linked to antigen presentation (*CD83*,* LAMP3*), migration to lymphoid tissues (*CCR7*, *ADAM19*), and the production of pro-inflammatory mediators (*TRAF1*,* IL24*,* ANXA1*) [28]. Dermatitis Herpetiformis (DH) is an autoimmune blistering skin condition linked to celiac disease (CD). Research has shown that *ADAM19* expression is elevated in DH patients. The role of *ADAM19* in DH may include facilitating the degradation of the extracellular matrix, aggravating skin barrier disruption, and influencing the inflammatory response, which collectively contribute to disease progression [29]. In patients with inflammatory bowel disease (IBD), *ADAM19* is overexpressed in inflamed intestinal tissues. Inflammatory cytokines can increase *ADAM19* production, thereby exacerbating the progression of the disease. *ADAM19* is implicated in the shedding of membrane-bound TNF-α, indicating that it may play a crucial role in the pathogenesis of IBD [30].

However, the expression of *ADAM19* in DCs of T1DM and its effects on pancreatic β-cell function are yet to be fully understood. This study therefore aims to explore how increased *ADAM19* expression in DCs of T1DM affects pancreatic β-cell function through modulation of the immune microenvironment, offering new perspectives for immune-based therapies in T1DM.

This study highlights the potential role of *ADAM19* in the immune microenvironment of T1DM through comprehensive data analysis. By performing PLS-DA on four independent datasets (GSE156035, GSE55100, GSE9006, and in-house dataset), we identified significant transcriptomic differences between the control and T1DM groups. Intersection analysis further revealed three genes that were upregulated across all datasets: *ADAM19*, *HLX*, with *ADAM19* being selected as the primary focus of this study. Statistical analysis indicated that *ADAM19* expression was significantly higher in the T1DM group compared to the control group, with consistent upregulation observed across all datasets. ROC curve analysis demonstrated that *ADAM19* effectively distinguishes T1DM patients from healthy controls, with AUC values exceeding 0.7 across all datasets. Remarkably, in the in-house dataset, the AUC reached 0.93, highlighting its strong diagnostic performance. This further underscores its potential as a valuable biomarker.

ScRNA-seq analysis identified a marked upregulation of *ADAM19* in DCs, which was further confirmed by UMAP plots, showing its enrichment in the T1DM immune microenvironment. This suggests that *ADAM19* may play a critical role in regulating the function of DCs. We observed that *ADAM19* expression increased gradually along the trajectory (Fig. 3F-G). By mapping the trajectory across different subtypes, we observed that *ADAM19* expression increased during subtype transitions. Additionally, *ADAM19* expression was elevated as the progression shifted from normal to disease states. Thus, elevated ADAM19 expression is associated with both disease development and subtype transitions(Figure 3 H-K). To further validate our findings, we expanded the sample size, and the results revealed that *ADAM19* expression was significantly elevated in T1DM patients compared to normal controls. Moreover, patients with higher *ADAM19* expression exhibited notably lower fasting C-peptide levels, indicating a potential link between *ADAM19* and impaired pancreatic islet function in T1DM patients.

Based on association analysis, we speculate that *ADAM19* may contribute to the onset and progression of T1DM in the following ways: First, *ADAM19* may be involved in the shedding of membrane-bound tumor necrosis factor-alpha (TNF-α), thereby activating immune cells, exacerbating local inflammatory responses, and amplifying autoimmune attacks. Second, the elevated expression of *ADAM19* in DCs may enhance antigen presentation and cytokine production, influencing T cell activation and differentiation, and further driving the autoimmune response of the immune system. In addition, the differential expression of *ADAM19* across DC subsets suggests that it may play an important role in shaping the immune microenvironment and in the immune dysregulation associated with T1DM. Finally, overexpression of *ADAM19* may also regulate the degradation of the extracellular matrix, compromising the integrity of the islet barrier and further exacerbating β-cell damage and functional decline. Of course, the causal relationships proposed here still await further experimental validation and mechanistic investigation.

### Limitations and future perspectives

This study has several limitations. First, it primarily relies on transcriptomic and single-cell RNA sequencing data, future studies should incorporate larger clinical cohorts and include protein-level validation (e.g., flow cytometry). Second, although cell–cell communication analysis suggests that *ADAM19* may be involved in immune regulation, the precise mechanisms remain to be elucidated through animal models and clinical intervention studies. Third, pseudotime analysis represents computational predictions rather than actual cellular trajectories. Moreover, *ADAM19* exhibits considerable overlap in expression ranges between groups and is therefore not suitable as a standalone diagnostic marker, but may serve as a complementary marker in multi-marker panels alongside existing autoantibody tests.

Additionally, all 604 DCs analyzed in our single-cell RNA-seq dataset were derived from PBMCs, despite the inclusion of pancreatic islet samples from the HPAP cohort. Consequently, our findings primarily reflect systemic changes in circulating DCs rather than those within the insulitic lesions in the pancreas. An alternative explanation for the observed upregulation of *ADAM19* in peripheral DCs could be differential homing or migration of *ADAM19*-expressing DCs to the pancreatic islets in T1DM. However, several lines of evidence argue against this being the sole explanation: (1) *ADAM19* itself is significantly upregulated within DCs (fold change = 1.78, adjusted *p* = 0.008); (2) cell–cell communication analysis revealed strengthened *FURIN*-*ADAM19* interactions between DCs and pancreatic resident cells in T1DM; (3)Published rheumatoid arthritis data [28] show that under TNF stimulation, *ADAM19* and *CCR7* (a key chemokine receptor for DC migration) are co-upregulated. This indicates that DCs with high *ADAM19* expression have enhanced migratory capacity rather than remaining as sessile cells in the periphery. and (4) elevated peripheral *ADAM19* expression is significantly associated with reduced fasting C-peptide levels.

Future prospective multi-center studies combining paired PBMC-pancreas sampling or spatial transcriptomics are needed to validate the clinical utility of *ADAM19* as a biomarker and to clarify its local versus systemic roles in the pancreatic immune microenvironment.

## Conclusions

In this study, we found that *ADAM19* expression is significantly elevated in DCs of patients with T1DM. This upregulation was consistently observed across multiple independent bulk RNA-seq datasets as well as our in-house cohort, and was further confirmed by single-cell RNA-seq analysis of both PBMCs and pancreatic tissues. ROC analysis demonstrated that *ADAM19* exhibits good discriminatory power as a candidate biomarker for T1DM, with AUC values ranging from 0.71 to 0.93.

Single-cell analysis revealed that elevated *ADAM19* expression is closely associated with shifts in DC subset dynamics, enhanced cell–cell communication (particularly through the *FURIN-ADAM19* axis), and immune microenvironment dysregulation. Notably, higher *ADAM19* levels in peripheral blood were significantly correlated with impaired pancreatic β-cell function, as evidenced by markedly reduced fasting C-peptide levels in patients with high *ADAM19* expression.

These findings suggest that *ADAM19* may contribute to immune dysregulation and β-cell damage in T1DM by promoting DC activation, migration, and pro-inflammatory signaling, including TNF-α shedding. This is consistent with previous observations in rheumatoid arthritis, where TNF stimulation co-upregulates *ADAM19* and the key migration receptor *CCR7*, indicating that *ADAM19*-high DCs possess enhanced migratory capacity rather than remaining sessile in the periphery.

Taken together, our results identify *ADAM19* as a promising candidate biomarker for T1DM and highlight its potential as a therapeutic target, warranting further mechanistic investigations and clinical validation.In summary, this study identifies elevated *ADAM19* in DCs associated with T1DM immune features and β-cell dysfunction markers. As a candidate biomarker, it warrants further mechanistic and clinical validation.

## Electronic Supplementary Material

Below is the link to the electronic supplementary material.

Supplementary Material 1

Supplementary Material 2: Fig S1 Cell-type specificity of ADAM19 expression and compositional analysis in T1DM patients versus healthy controls. (“All samples/all cell types” refers to the integrated single-cell RNA-seq dataset combining PBMC and pancreatic tissue samples, encompassing epithelial, immune, and stromal cells; “PBMC” refers exclusively to peripheral blood mononuclear cell samples containing only immune cell subsets.) A. Percentage of cells expressing ADAM19 across major cell types in the integrated dataset (% Cells Expressing ADAM19). B. Proportions of different cell types in pancreatic tissue from healthy controls versus T1DM patients. C. Proportions of different cell types in PBMC samples from healthy controls versus T1DM patients. D. ADAM19 expression levels within each cell type in the integrated dataset (T1DM vs. healthy controls). Violin plots show expression distributions with annotated p-values. E. ADAM19 expression levels in major immune cell subsets in PBMC samples only (T1DM vs. healthy controls). Violin plots show expression distributions with annotated p-values.

## Data Availability

The dataset(s) supporting the conclusions of this article are available upon request from the corresponding author via email.
