# Supplementary material for: Upregulation of ADAM19 in dendritic cells is associated with immune features and lower C-peptide levels in type 1 diabetes mellitus
Source: Diabetol Metab Syndr. 2026 Jun 5;18:172. doi: 10.1186/s13098-026-02170-8 (PMC13459314; doi:10.1186/s13098-026-02170-8)
Supplement: Supplementary file 1 [file 13098_2026_2170_MOESM1_ESM.pdf]

Figure S1

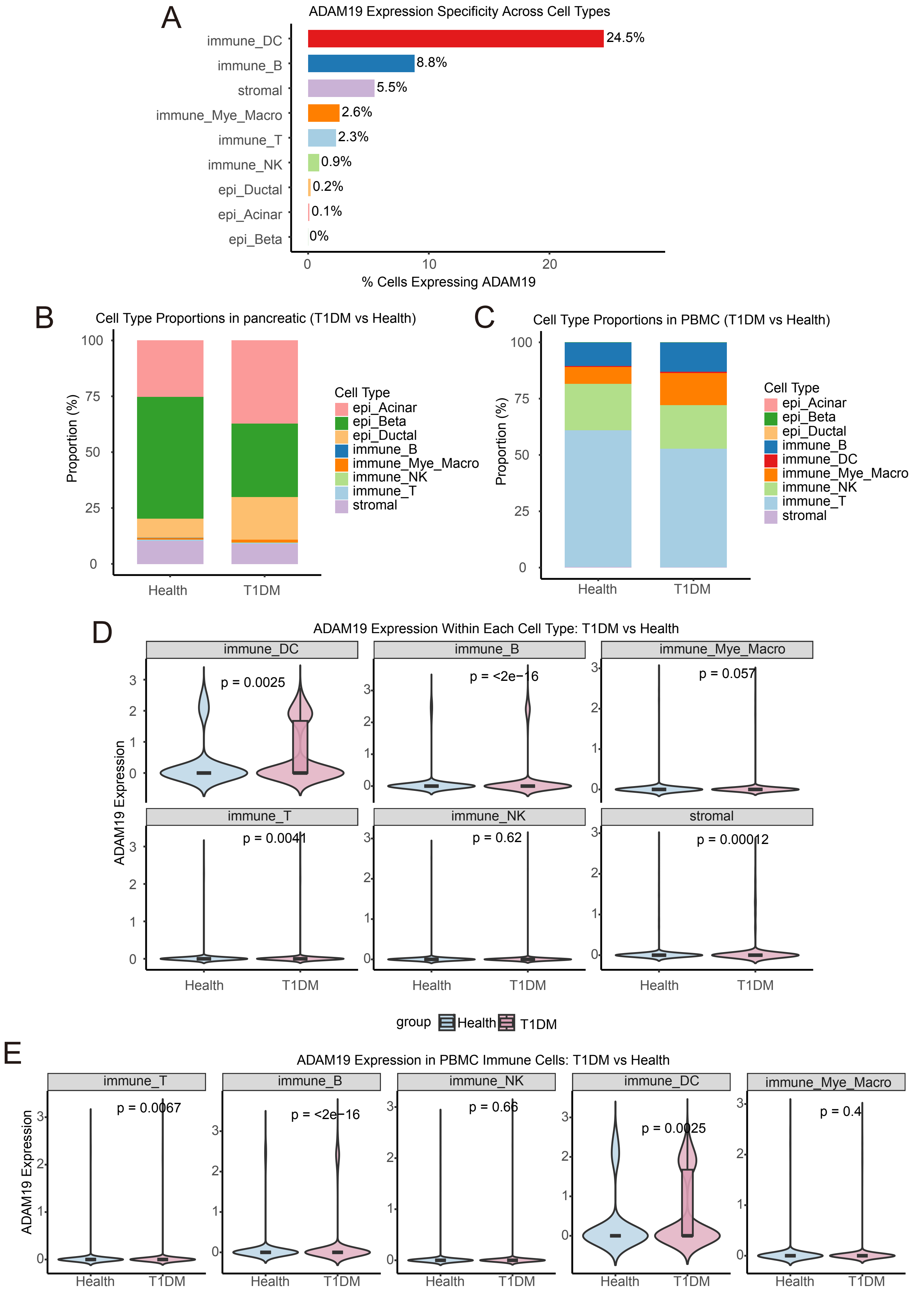
A
ADAM19 Expression Specificity Across Cell Types
immune_DC
immune_B
stromal
immune_Mye_Macro
immune_T
immune_NK
epi_Ductal
epi_Acinar
epi_Beta
24.5%
8.8%
5.5%
2.6%
2.3%
0.9%
0.2%
0.1%
0%
0
10
20
% Cells Expressing ADAM19
B
Cell Type Proportions in pancreatic (T1DM vs Health)
Proportion (%)
100
75
50
25
0
Health
T1DM
Cell Type
epi_Acinar
epi_Beta
epi_Ductal
immune_B
immune_Mye_Macro
immune_NK
immune_T
stromal
C
Cell Type Proportions in PBMC (T1DM vs Health)
Proportion (%)
Health
T1DM
Cell Type
epi_Acinar
epi_Beta
epi_Ductal
immune_B
immune_DC
immune_Mye_Macro
immune_NK
immune_T
stromal
D
ADAM19 Expression Within Each Cell Type: T1DM vs Health
immune_DC
p = 0.0025
immune_B
p = <2e−16
immune_Mye_Macro
p = 0.057
immune_T
p = 0.0041
immune_NK
p = 0.62
stromal
p = 0.00012
ADAM19 Expression
Health
T1DM
group
Health
T1DM
E
ADAM19 Expression in PBMC Immune Cells: T1DM vs Health
immune_T
p = 0.0067
immune_B
p = <2e−16
immune_NK
p = 0.66
immune_DC
p = 0.0025
immune_Mye_Macro
p = 0.4
ADAM19 Expression
Health
T1DM
